# ACE Insertion/Deletion Polymorphism and Diabetic Nephropathy: Clinical Implications of Genetic Information

**DOI:** 10.1155/2014/846068

**Published:** 2014-12-23

**Authors:** Sung-Kyu Ha

**Affiliations:** Renal Division, Department of Internal Medicine, Gangnam Severance Hospital, Yonsei University College of Medicine, Dogok-Dong, Gangnam-Gu, Seoul 146-92, Republic of Korea

## Abstract

Approximately 20–40% of diabetic patients develop nephropathy which is the leading cause of ESRD in developed countries. The ACE I/D polymorphism is thought to be a marker for functional polymorphism which regulates circulating and tissue ACE activity. While the initial study found a protective effect of the II genotype on the development of nephropathy in IDDM patients, subsequent studies have addressed the role of ACE I/D polymorphism in the development and progression of diabetic nephropathy. RAAS blockers are the first line drugs for the treatment hypertension associated with diabetes and have been widely used in everyday clinical practice for the purpose of reducing proteinuria in patients with various renal diseases. However, the antiproteinuric effect of RAAS blockers is variable and the percentage of reducing proteinuria is in the range of 20–80%. The antiproteinuric effect of RAAS blockers may be related to a number of factors: the type or the dose of RAAS blockers, the duration of therapy, the level of sodium intake, and the type of patient's ACE I/D genotype. Besides the nongenetic factors, drug responses, can be influenced by ACE gene polymorphism. In this review, we discuss the relationship between ACE I/D polymorphism and diabetic nephropathy and therapeutic response of RAAS blockers.

## 1. Introduction

Chronic kidney disease (CKD) is a worldwide public health problem that affects millions of people from all over the world. Diabetic nephropathy is a common complication in patients with diabetes and the leading cause of end-stage renal disease (ESRD) [1]. Over the last decade, the prevalence of diabetes has increased worldwide, as a result of the continuous rise in type 2 diabetes incidence [2]. Approximately 20–40% of diabetic patients including IDDM and NIDDM develop diabetic nephropathy. The pathogenesis of this drastic complication is not clearly understood, but available data suggests that multiple factors such as hemodynamic alterations, metabolic abnormalities, various growth factors, and genetic factors contribute to the pathogenesis of diabetic nephropathy. In experimental and human diabetic nephropathy, systemic and glomerular hypertension played a role in the initiation and progression of nephropathy [3]. These hemodynamic changes may be explained in part by alterations in the renin-angiotensin system. Consequently, genes involved in the renin-angiotensin system have been suggested as potential genetic predispositions for the development of diabetic nephropathy. Many previous publications suggest that genetic predisposition plays a role in the development of diabetic nephropathy which clusters within families, both in type 1 (IDDM) and type 2 (NIDDM) diabetes mellitus [4–7]. Polymorphism means the phenomenon of having two or more genetic variants. DNA polymorphism, so far, is known to exist for the great majority of human genes. In diabetes mellitus, several models can be figured out to represent different concepts of the pathogenesis of diabetic nephropathy and to incorporate genetic factors [8]. Angiotensin-I converting enzyme (ACE) is one of the key enzymes in the renin-angiotensin-aldosterone system (RAAS) and the insertion (I)/deletion (D) polymorphism of this gene has been studied extensively with renal [9] and cardiovascular [10] complications of diabetic nephropathy.

## 2. Structure of ACE Gene and Its Insertion/Deletion Polymorphism

The ACE, which was originally discovered in equine plasma, is a membrane-bound dipeptidyl carboxypeptidase ectoenzyme located in the endothelial lining of blood vessels throughout the body where it plays an important role in proliferation of vascular smooth muscle cells through the conversion of angiotensin-I to angiotensin II and bradykinin inactivation [11]. The ACE is found as a membrane-bound enzyme in endothelial cells and different types of epithelial and neuroepithelial cells as well as in circulating form in biological fluids, such as plasma, cerebrospinal fluid, amniotic fluid, and seminal fluids [11, 12]. The mechanisms that lead to the biosynthesis of the circulating form of ACE are unclear, but available data indicates that its structure is very similar to that of the cellular form. The circulating form is virtually identical to the cellular form except for the lack of the transmembrane and intracellular sequence but whether it arises from specific proteolytic cleavage from the cell surface or nonspecifically from senescent cells has yet to be determined [12]. ACE genes have been cloned in the human, mouse, and rabbit and the enzyme was the product of one gene in man [12, 13]. The human and mouse genes were made up of 26 exons and 25 introns. Most somatic forms of ACE appear to be transcribed from all exons except exon 13 which encodes the unique N-terminal region of the testicular form [12]. The human ACE gene was localized to the long arm of chromosome 17. Cloning of the ACE gene had made it possible to identify a 287 bp insertion/deletion polymorphism in intron 16 (ACE I/D polymorphism) that appears to affect the level of serum ACE activity. The genotype with deletion of the 287 base pair resulted in higher plasma ACE levels [14]. The plasma ACE was predominantly derived from tissue vascular endothelial cells suggesting that patients with the DD genotype might have higher tissue angiotensin II [11]. Angiotensin II might modulate the growth of smooth muscle cell and induce myointimal hyperplasia after endothelial injury [15], and administration of ACE inhibitors prevented myointimal proliferation after vascular injury [16].

## 3. Genetic Control of Plasma ACE Level

Plasma ACE concentrations are stable when measured repeatedly in a normal subject [17] but large interindividual variations make it difficult to interpret plasma ACE levels in a given patient [11]. The study conducted in a large sample of healthy families showed an intrafamilial resemblance between ACE levels and also suggested that they were subject to the effect of major gene [18]. The ACE I/D polymorphism accounts for over 40% of interindividual variability of serum or tissue ACE activity [19]. Patients homozygous for the deletion had the greatest serum ACE activity, whereas those homozygous for the insertion had the lowest level. An insertion/deletion (I/D) polymorphism of intron 16 of the angiotensin-I converting enzyme (ACE) gene is largely responsible for variations in plasma ACE levels and for explaining approximately 50% of the variability in serum ACE activity observed [14]. Using the linkage-segregation analysis of the plasma ACE, Cambien [11] has shown that the ACE I/D polymorphism was a marker for an unknown functional polymorphism (ACE S/s) which appeared to be a new independent risk factor for myocardial infarction.

## 4. ACE Insertion/Deletion Polymorphism and the Development of Diabetic Nephropathy

The ACE I/D polymorphism is thought to be a marker for functional polymorphism which regulates circulating and tissue ACE activity [14, 18, 19]. Since 1990, its association with diabetic nephropathy has been extensively investigated and more than 300 studies have explored genetic associations of this polymorphism in more than 100 conditions including diabetic nephropathy [20]. Up to now, the I/D polymorphism of ACE gene is the most extensively studied marker for association with diabetic nephropathy. While the initial study by Marre et al. [21] found a protective effect of the II genotype on the development of nephropathy in IDDM patients, subsequent studies have yielded inconsistent results. Over the past decades, many studies have been conducted to examine the association between ACE I/D polymorphism and nephropathy in type 1 and type 2 diabetes, but the results still remain inconsistent. However, recently conducted meta-analysis showed consistent association between ACE D allele or DD genotype and ESRD risk in diabetic nephropathy patients. In 2005, Ng et al. [22] reported a meta-analysis of 14727 diabetic patients including 47 studies (8,663 cases, 6,064 controls) from 1994 to 2004. They confirm a statistically significant protective role of the II genotype in the development of diabetic nephropathy; the effect was most pronounced in Asians with type 2 diabetes, followed by Caucasians with types 1 and 2 diabetes. They suggested that these findings may have implications for the management of diabetic nephropathy using ACE inhibitors especially among type 2 diabetic Asians. In 2012, another meta-analysis was reported by Wang et al. [23]. They conducted a comprehensive meta-analysis on 63 published studies from 1994 to 2010 with 14,108 cases and 12,472 controls relating variants of the ACE I/D polymorphism to the risk of developing diabetic nephropathy. They included all of the studies that determined the genotype distribution of ACE I/D polymorphism in cases with type 1 or type 2 diabetes and nephropathy and in diseased controls or in healthy controls. The overall analysis showed a significant association between the* ACE* I/D polymorphism and the risk of diabetic nephropathy for all genetic models (ID versus II: OR = 1.12, 95% CI 1.02–1.24; DD versus II: OR = 1.27, 95% CI 1.13–1.44; allele contrast: OR = 1.15, 95% CI 1.08–1.23; dominant model: OR = 1.18, 95% CI 1.07–1.31; and recessive model: OR = 1.18, 95% CI 1.08–1.30, resp.). In stratified analysis by ethnicity and diabetes mellitus type, they further found that the Asian group with type 2 DM showed a significant association for all genetic models (ID versus II: OR = 1.25, 95% CI 1.07–1.47; DD versus II: OR = 1.57, 95% CI 1.24–1.98; allele contrast: OR = 1.30, 95% CI 1.15–1.46; dominant model: OR = 1.37, 95% CI 1.10–1.69; and recessive model: OR = 1.34, 95% CI 1.15–1.56, resp.). However, they failed to find any significant effects for different genetic models in other subgroups. The authors suggested that the ACE I/D polymorphism may contribute to nephropathy development, especially in the Asian group with type 2 diabetes mellitus.

## 5. ACE Insertion/Deletion Polymorphism and the Progression of Diabetic Nephropathy to ESRD

In 1996, Yoshida et al. [24] including 168 Japanese patients with NIDDM followed over 10 years have shown that ACE DD genotype has a high prognostic value for progressive deterioration of renal function. They also showed that patients with stable renal function had no significant difference in the distribution of ACE genotype between patients with albuminuria and patients without albuminuria. Analysis of the clinical course of the three ACE genotypes revealed that the majority (95%) of patients with the DD genotype who had albuminuria progressed to end-stage renal disease within 10 years of diagnosis of diabetes. Moreover, the ACE DD genotype had the increased mortality in patients on dialysis and the prevalence of the DD genotype in patients on chronic dialysis decreases year by year. Next year, Schmidt et al. [25] reported 658 Caucasian patients with type II diabetes, 347 without diabetic nephropathy and 311 with various stages of diabetic nephropathy, and determined the I/D polymorphism of the ACE gene. They compared patients at the extremes of renal risk, that is, normotensive patients without antihypertensive treatment and without nephropathy (*n* = 144) versus patients on dialysis (*n* = 61), differed with respect to genotype (DD 36.8% versus 57.4%; *P* = 0.007) and allele frequencies (D 0.59 versus 0.76; *P* < 0.001). In this study, patients with the highest renal risk more frequently had the DD genotype. This would be compatible with a greater risk of (or rate of) progression to end-stage renal failure. Vleming et al. [26] also compared the ACE genotype distributions in 79 Caucasian IDDM patients with ESRD and 82 control patients without microalbuminuria after fifteen years of IDDM. The ACE genotype distribution in patients was not in accordance with the Hardy-Weinberg equilibrium due to a significant overrepresentation of the DD genotype (*χ* ^2^ = 8.9, *P* = 0.01). The presence of the DD genotype increased the risk of end-stage renal failure two-fold compared to the other genotypes (odds ratio 2.1, 95% CI 1.1–4.0). They concluded that the risk of end-stage renal failure in patients with IDDM is two-fold increased in patients with the DD genotype as compared to patients with other genotypes. We also studied the impact of insertion/deletion (I/D) genotypes on the progression of diabetic nephropathy in 239 Korean patients with type 2 diabetes (Group 1, 99 patients with stable renal function; Group 2, 140 patients with declining renal function) [27]. The frequency of the DD genotype was significantly greater in group 2 compared with group 1 (30.7% versus 9.1%; *P* < 0.05). Patients with the DD genotype reached the end point (serum creatinine > 2.0 mg/dL [176.8 micromol/L]) faster than those with the other genotypes (DD, 11.38 ± 4.08 years; ID, 13.85 ± 4.04 years; II, 14.04 ± 4.06 years, resp., *P* < 0.05) and took significantly less time to reach dialysis therapy (DD, 13.10 ± 4.45 years; ID, 16.21 ± 4.74 years; II, 15.13 ± 4.09 years, resp., *P* < 0.05). In multiple logistic regression analysis, systolic blood pressure and DD genotype showed significant correlations with the progression of diabetic nephropathy. In patients with the DD genotype, the odds ratio was 3.881 (95% confidence interval, 1.564 approximately 9.628; *P* = 0.003) compared with that with the II genotype. We suggested that the ACE DD genotype might be a significant risk factor for the progression of diabetic nephropathy. Recently, Yu et al. [28] published meta-analysis of 12 previous studies containing 4,015 diabetic nephropathy patients (981 cases, 3,034 controls). In this study, they showed that in overall populations, there was a notable association between D allele or DD genotype and ESRD susceptibility (D: OR 1.32, 95% CI 1.11–1.56, *P* = 0.002; DD: OR 1.67, 95% CI: 1.25–2.21, *P* = 0.004). In the subgroup analysis according to ethnicity, D allele or DD genotype was associated with ESRD risk in Asians. In Caucasians, the association of DD genotype with ESRD risk was observed, but the D allele was not. Furthermore, ACE I/D gene polymorphism was associated with ESRD risk in patients with diabetic nephropathy due to type 2 DM, but the association was not found for patients with diabetic nephropathy due to type 1 DM. They concluded that D allele or DD genotype is associated with the ESRD susceptibility in diabetic nephropathy patients.

## 6. ACE I/D Polymorphism and Antiproteinuric (Renoprotective) Responses to RAAS Inhibitor Therapies in Diabetic Nephropathy

Earlier studies repeatedly showed that patients with DD genotype or D allele have elevated circulating and tissue ACE activity [14, 19] compared to patients with I allele. This may contribute to the interindividual variability in the antiproteinuric responses to inhibition of the RAAS using either ACE inhibitors (ACEIs) or angiotensin II type 1 receptor blockers (ARBs) [29]. RAAS blockers are the first-line drugs for the treatment of hypertension associated with diabetes in the fact that these drugs not only lower systemic blood pressure but also reduce intraglomerular pressure. Imanishi et al. [30] showed that the mechanism by which an ACE inhibitor caused a short-term decrease in albuminuria in early diabetic nephropathy involved a glomerular hemodynamic change, namely, a decrease in intraglomerular capillary pressure. These drugs have greatly improved the renal prognosis and survival of diabetic patients with nephropathy over the last decade [31]. However, despite therapy targeting elevated blood pressure, albuminuria, hyperglycemia, and lipid abnormalities, patients with diabetic nephropathy still on average have a rate of decline in renal function three to six times that seen in individuals without renal disease [31, 32]. Consequently, diabetic nephropathy is still one of the principal causes of end-stage renal disease, leading to dialysis and death in developed countries. Although current therapeutic strategies have alleviated the huge burden of diabetic nephropathy, many patients still progress to ESRD. One reason for the inadequacy of current antiproteinuric (renoprotective) therapy and the persistent poor renal prognosis is the large interindividual variation in response to first-line therapy including antihypertensive drugs blocking the renin-angiotensin-aldosterone system [33]. Furthermore, overt proteinuria seen in diabetic nephropathy is itself a risk factor that may adversely affect renal function, and it is associated with a faster rate of renal disease progression. Therefore, the reduction of proteinuria is an important tool for retarding the progression of renal disease in diabetic nephropathy patients [34]. Traditionally, ACE inhibitors (ACEIs) and angiotensin II type 1 receptor blockers (ARBs) have been widely used in everyday clinical practice of nephrology for the purpose of reducing proteinuria in patients with various renal diseases including diabetes mellitus. However, the antiproteinuric effect of ACE inhibitors on proteinuria is variable and the percentage of reducing proteinuria is in the range of 20–80% in a variety of renal diseases [35–37]. The antiproteinuric effect of ACE inhibitors and/or angiotensin II type 1 receptor blockers may be related to a number of factors: the type or dose of the RAAS blockers, the duration of therapy, the level of sodium intake, and the type of patient's ACE genotype [29, 38–40]. The antiproteinuric mechanisms of RAAS blockers are thought to decrease intraglomerular capillary pressure by reducing both afferent and efferent renal arteriolar resistance, predominantly dilating efferent arteriole and systemic blood pressure [41, 42]. As demonstrated in previous studies, RAAS blockade has been superior to other antihypertensive agents in reducing albuminuria and slowing rate of decline in GFR despite similar blood pressure controls. Besides the nongenetic factors, drug responses are also influenced by inherited factors such as ACE gene polymorphism. The basic concept of pharmacogenomics is a drug interacting with its target, for instance, an enzyme or a receptor. When genetic polymorphism leads to modified target availability or function, the drug response is modified as well [43]. It has been reported that ACE I/D genotypes appeared to be a major determinant of plasma and tissue ACE activities [14, 18, 19]. Individuals with the DD genotype have the greatest and those with II genotype have the least ACE concentrations. So, it is expected that the differences in plasma and tissue ACE activities associated with ACE I/D genotype might affect the antiproteinuric (renoprotective) responses to RAAS inhibition. Several studies concerning antiproteinuric (renoprotective) effect of RAAS blockers and ACE I/D polymorphism in diabetic nephropathy were reported so far [34, 44–53]. However, the antiproteinuric (renoprotective) effect of RAAS blockers and ACE I/D genotype in diabetic nephropathy is inconclusive. These results may be due to incomplete blockade of RAAS by suboptimal doses and/or compensatory mechanisms occurring during long-term treatment with RAAS blockers. After initiation of ACEI treatment, angiotensin II level in plasma is lowered initially. However, during long-term treatment of ACEI, angiotensin II level tends to increase as a result of ACE escape and angiotensin II generation through non-ACE dependent pathways such as chymase or other serine proteases. Incomplete RAAS blockade during chronic ACEI therapy may be overcome by inhibiting the action of angiotensin II at the site of the angiotensin II type 1 receptor by an ARB. On the contrary, in treatment with ARB, there also tends to be a compensatory increase in renin and angiotensin II levels, thereby increasing the competition at the angiotensin II type 1 receptor site. So, in this situation, we can reduce compensatory increased angiotensin II by adding ACEI.

### 6.1. ACE Inhibitors and ACE I/D Polymorphism

Several previous studies suggested that ACE genotype may predict the response of patients to antiproteinuric and renoprotective effect with ACE inhibitors (ACEIs). Parving et al. [44], in their observational followup study of type 1 diabetic patients with diabetic nephropathy receiving ACE inhibitor captopril (*n* = 35 and *n* = 169), have reported that the DD genotype reduces the long-term beneficial effect of ACE inhibition on the progression of diabetic nephropathy in patients with IDDM. Jacobsen et al. [45] tested the potential role of ACE I/D polymorphism on the early antiproteinuric responsiveness in an observational followup study with sixty young hypertensive type 1 DM nephropathy patients. They showed more albuminuria reduction with captopril therapy in II genotype than in ID or in DD genotypes. Data from a EURODIAB randomized controlled trial lisinopril in IDDM [46] showed that the I/D polymorphism of the ACE gene modulates the therapeutic effect of ACE inhibition on the progression of urinary albumin excretion in IDDM patients. Patients with the II genotype showed the fastest rate of AER progression on placebo but had an enhanced response to lisinopril. Albumin excretion rate (AER) at 2 years (adjusted for baseline AER) was 51.3% lower on lisinopril than placebo in the II genotype patients (95% CI, 15.7 to 71.8; *P* = 0.01), 14.8% lower in the ID group (–7.8 to 32.7; *P* = 0.2), and 7.7% lower in the DD group (–36.6 to 37.6; *P* = 0.7). Only in the II group the difference was statistically significant even after adjustment for gender, baseline and followup BP, and metabolic control. The authors concluded that knowledge of ACE genotype may be of value in determining the likely impact of ACE inhibitor treatment on the albuminuria reduction. Another study from Jacobsen et al. [47], which included 169 IDDM patients with overt nephropathy, showed that the D allele of the ACE I/D polymorphism in addition to nongenetic risk factors independently accelerated progression of diabetic nephropathy during ACE inhibition. Also, in this study of patients with type 1 diabetes, the I allele was associated with a slower progression to doubling of serum creatinine or ESRD. Taken together, in type 1 diabetes with nephropathy, the I allele increases the responsiveness to the antiproteinuric (renoprotective) effect of ACE inhibitor therapy. Data in type 2 DM nephropathy in relation to ACE I/D polymorphism and antiproteinuric (renoprotective) effect of ACE inhibitors are scarce. Our group investigated the antiproteinuric effect of ACE inhibition (benazepril 10 mg/day or perindopril 4 mg/day) in relation to ACE I/D polymorphism in a short-term observational followup study in 83 NIDDM patients with overt nephropathy (urinary protein excretion over 500 mg/day) classified into three groups in accordance with ACE genotypes (17 DD; 33 ID; 33 II) and prospectively followed up for 3 months [34]. Before entry, previously used ACE inhibitors were withdrawn for at least 2 weeks and baseline proteinuria was measured. Our study showed that the percentage reduction in proteinuria for DD genotype was significantly higher than in ID and in II genotypes (50.9 ± 19.2% versus 19.2 ± 16.0%, 20.2 ± 20.4%, *P* < 0.05). There were no statistically significant correlations between the levels of baseline proteinuria and the magnitudes of the reduction of proteinuria under ACE inhibition (*P* > 0.05). These results indicated that ACE DD genotype is more susceptible than ACE II and ID genotypes to the antiproteinuric effect of ACE inhibitors. However, this investigation had small subjects and short-term followup period. Another study by So et al. [48] provided more evidence supporting the importance of II genotype in response to ACE inhibition. They found that, in 2089 Chinese patients with normoalbuminuria, microalbuminuria, or macroalbuminuria over a median period of 44.6 months, ACE inhibitor therapy decreased mortality and renal end point which was defined as death due to renal failure, dialysis, eGFR of <15 mL/min/1.73 m^2^ or more than 50% loss of eGFR, more effectively in I allele carriers than in DD. This study was the only study in type 2 diabetes with adequate power and followup. However, we have to be cautious in interpreting this finding because the favorable effects of the I allele were restricted to those with normoalbuminuria or microalbuminuria. Another study regarding RAAS gene polymorphisms and renal responsiveness to RAAS inhibition in type 2 diabetic Asian Indians was reported recently by Cheema et al. [49] from India. They enrolled 810 north Indian type 2 diabetics treated with ACE inhibitor or ARB and were followed up for 3 years. They observed that the ACE II genotype and cumulative genetic risk score of < 1 was associated with better renoprotective response to ACE inhibitor in type 2 DM with normoalbuminuria. However, there was no significant difference in renoprotective effect in type 2 diabetics with nephropathy based on ACE I/D genotypes with 3-year ACE inhibitor therapy (Table 1).

### 6.2. Angiotensin II Type 1 Receptor Blockers and ACE I/D Polymorphism

Data on angiotensin II type 1 receptor blocker (ARB) in relation to ACE I/D polymorphism and antiproteinuric (renoprotective) effects in type 1 diabetes with nephropathy are also scarce. Two small studies by Andersen et al. [50, 51] were found in the literatures. They showed that the antiproteinuric effect of 36- and 4-month ARB (losartan) had similar beneficial renoprotective and antiproteinuric effect in 54 hypertensive white type 1 DM nephropathy patients with ACE II and DD genotypes. Same result was reported by Haneda et al. [52] in 127 Japanese type 2 DM nephropathy patients. They found that ARB (candesartan) is useful in reducing proteinuria in type 2 DM nephropathy patients compared with placebo and seems to be effective in subjects with both the II and DD genotypes of the ACE gene. More data on the interactions between ACE I/D polymorphism and ARB therapy have been provided by analyses of the Reduction of Endpoints in NIDDM with the AII Antagonist Losartan (RENAAL) study, a double blind, multicenter, prospective, randomized, and placebo controlled clinical trial designed to evaluate the renal effects of losartan in 1513 type 2 diabetic patients with overt nephropathy [53]. ACE I/D data were available in 1435 of the 1513 RENAAL study patients. Available ACE I/D data showed that compared with placebo losartan reduced the risk of reaching the composite end point of doubling of serum creatinine, ESRD, or death by 5.8%, 17.6%, and 27.9% among those with the II, ID, and DD genotypes, respectively. This study demonstrated that the deletion allele of the ACE gene had a harmful impact on the composite endpoint. The beneficial effects of losartan were greatest in the ACE DD genotype group and intermediate in the ACE ID genotype group for nearly all endpoints. The novel clinical importance of this study is that those patients who have the greatest need for renoprotective treatment have the best effect of losartan (DD and ID genotype), whereas those patients with a better renal prognosis (II genotype) also derived renal benefit. This is the largest, double blind randomized study with adequate statistical power. However, several limitations exist in this study. First, this is the largest trial evaluating the association of the ACE/ID polymorphism on renal outcome and death during angiotensin II-receptor blockade in diabetic nephropathy; the study may well be underpowered in relation to the individual components of composite endpoint. Second, the ID alleles were not in Hardy-Weinberg equilibrium in the black patients. Lastly, despite randomization, higher baseline proteinuria was present in the II genotype in the losartan group. In spite of the several limitations of this study, the deletion allele of the ACE I/D polymorphism showed unfavorable renal prognosis in patients with proteinuric type 2 diabetes, which can be improved by losartan treatment. Another recent study by Cheema et al. [49] also reported that, in type 2 diabetics with nephropathy, ACE DD genotype and a genetic risk score of >6 were associated with better renoprotective response to ARB. They suggested that ACE I/D genotypes individually and in interaction with other RAS single nucleotide polymorphisms (angiotensinogen and angiotensin II type 1 receptor gene) modulate renoprotective efficacy of ACE inhibitor and ARB in type 2 diabetics depending on the status of proteinuria (Table 2).

## 7. Conclusions

The association between the RAAS activation and the development and progression of diabetic nephropathy has been known for a long period of time. ACE is the key enzyme of the RAAS-cascade that plays a central role of blood pressure regulation and volume homeostasis in the body. Despite the large amount of studies looking for candidate genes, the ACE I/D polymorphism remains the unique and well-characterized locus clearly associated with development and progression of diabetic nephropathy. After the initial study by Marre et al. [21], numerous studies have addressed the role of ACE I/D polymorphism in the development and progression of diabetic nephropathy. Data reported so far showed that (1) the ACE I/D polymorphism directly influences circulating levels of ACE, (2) the II genotype protects against the development of diabetic nephropathy, (3) the DD genotype predicts poor renal response to RAAS inhibitors (the current strategy of RAAS inhibition in patients with the DD genotype may be insufficient to block an activated RAAS), and (4) angiotensin II type 1 receptor blockers (ARBs) can ameliorate the adverse effect of the D allele (no difference between genotypes is observed when patients are treated with an ARB). Evaluating the ACE I/D polymorphism in diabetic nephropathy is a reliable tool to identify diabetic patients at risk and identify patients who may benefit from antiproteinuric (renoprotective) therapy with ACE inhibitors and/or ARBs. This may guide pharmacologic therapy in individual patients and help to identify the patients with more aggressive use of RAAS blockers such as supramaximal dose of individual RAAS blocker and double or triple blockade of the RAAS. In case of an insufficient response to RAAS blockers, we also consider other treatment strategies such as glycemic control, low salt intake, more aggressive proteinuria reduction strategy, and hypercholesterolemia control that halt progression of diabetic nephropathy.

## Figures and Tables

**Table 1:** Studies examining the association of the ACE I/D polymorphism and response to antiproteinuric (renoprotective) effect of ACE inhibitor therapy.

Authors (year)	Ethnicity	Disease and patient number	Study durations (month)	Therapy drug	Effects on proteinuria or progression	Reference
Parving et al. (1996)	Caucasian	Type 1 DM (35)	84	Captopril	Faster progression and higher residual proteinuria in DD genotype	[44]
Jacobsen et al. (1998)	Caucasian	Type 1 DM (60)	6	Captopril	II genotype more albuminuria reduction	[45]
Penno et al. (1998)	Caucasian	Type 1 DM (530)	24	Lisinopril	II genotype more albuminuria reduction	[46]
Jacobsen et al. (2003)	Caucasian	Type 1 DM (169)	72	ACEIs (captopril, lisinopril, and enalapril)	D Allele accelerated progression of DMN	[47]
Ha et al. (2000)	Asian (Korean)	Type 2 DM (83)	3	Benazepril, perindopril	DD genotype more albuminuria reduction	[34]
So et al. (2006)	Asian (Chinese)	Type 2 DM (2089)	44.6	RAAS inhibitors	DD genotype higher risk of declining renal function	[48]
Cheema et al. (2013)	Asian (Indian)	Type 2 DM (490)	36	ACEIs	II genotype better renoprotective effect	[49]

ACE: angiotensin converting enzyme; ACEI: angiotensin converting enzyme inhibitor; ACE I/D polymorphism: angiotensin converting enzyme insertion/deletion polymorphism.

**Table 2:** Studies examining the association of the ACE I/D polymorphism and response to antiproteinuric (renoprotective) effect of ARB therapy.

Authors (Year)	Ethnicity	Disease and patient number	Study durations (month)	Therapy drug	Effects on proteinuria or progression	Reference
Andersen et al. (2002)	Caucasian	Type 1 DM (54)	4	Losartan	No differences in reduction of proteinuria	[50]
Andersen et al. (2003)	Caucasian	Type 1 DM (54)	36	Losartan	No differences in reduction of proteinuria	[51]
Haneda et al. (2004)	Asian (Japanese)	Type 2 DM (127)	3	Candesartan	No differences in reduction of proteinuria	[52]
Parving et al. (2008)	Mixed	Type 2 DM (1435)	40.8	Losartan	DD genotype more risk reduction reaching ESRD	[53]
Cheema et al. (2013)	Asian (Indian)	Type 2 DM (320)	36	ARBs	DD genotype associated with better renoprotective response	[49]

ARB: angiotensin receptor blocker; ACE I/D polymorphism: angiotensin converting enzyme insertion/deletion polymorphism.
